# On the role of the ventral attention system in spatial orienting

**DOI:** 10.3389/fnhum.2014.00235

**Published:** 2014-04-21

**Authors:** Ana B. Chica, Alexia Bourgeois, Paolo Bartolomeo

**Affiliations:** ^1^Department of Experimental Psychology, Brain, Mind, and Behaviour Research Center, University of GranadaGranada, Spain; ^2^Laboratory for Behavioral Neurology and Imaging of Cognition, Neuroscience Department, University of GenevaGeneva, Switzerland; ^3^INSERM UMRS 1127, Institut du Cerveau et de la Moelle Epinière et Université Pierre et Marie Curie, Groupe Hospitalier Pitié-SalpêtrièreParis, France; ^4^UPMC, Université Paris VIParis, France; ^5^Department of Psychology, Catholic UniversityMilan, Italy

**Keywords:** attention, fronto-parietal networks, ventral, dorsal, endogenous, exogenous

Macaluso and Doricchi (2013) carefully reviewed the functional Magnetic Resonance Imaging (fMRI) evidence on the dichotomy between dorsal and ventral fronto-parietal attentional networks, whose functioning has often been related to the behavioral differences between endogenous/strategic and exogenous/stimulus-driven attentional control, respectively (Corbetta et al., 2008). Contrary to this view, Macaluso and Doricchi (2013) emphasize that according to the available evidence either network can participate to both modes of orienting.

It is also proposed that a mere bottom-up stimulus onset is insufficient to activate the ventral network (Kincade et al., 2005; Indovina and Macaluso, 2007). Instead, bottom-up stimulus activation has to be matched to internal goals/expectations for the activation of the ventral network (see also Corbetta et al., 2008). Indeed, there is increased blood-oxygen-level dependent (BOLD) response in the ventral network for invalid vs. valid targets when attention is oriented using an endogenous, spatially predictive central cue, but not for invalid vs. valid targets preceded by an exogenous, spatially non-predictive peripheral cue (Natale et al., 2009). Moreover, Kincade et al. (2005) found no modulation of BOLD response in the ventral network during the cue-target interval, when attentional orienting occurs. This suggested that the sole dorsal attentional system regulates both exogenous and endogenous orienting. The ventral network would instead be implicated in reorienting to behaviorally relevant targets (Hahn et al., 2006; Corbetta et al., 2008). It is therefore proposed that the ventral network does not process task-irrelevant and non-predictive stimuli (which pertain to the traditional concept of exogenous attention). The ventral network would instead be involved in stimulus-driven updating of spatial expectations, but only when a task-relevant target or a set-relevant cue signals a “new” location that is potentially relevant.

Here we would like to suggest some caution in the interpretation of these results. The supporting evidence essentially comes from studies assessing variations in BOLD responses, whose temporal resolution is in the order of seconds. However, exogenous orienting is characterized by facilitatory components which peak at ~100 ms after cue onset (Müller and Rabbitt, 1989), and a subsequent Inhibition of Return (IOR), which is observed from ~300 ms after cue onset (Klein, 2000). We recently used a more time-resolved technique, Transcranial Magnetic Stimulation (TMS), to explore the functional contribution of attentional regions during exogenous attentional orienting elicited by non-predictive peripheral cues (Chica et al., 2011). During the cue-target interval of a typical Posner task (Chica et al., 2014), we delivered two TMS pluses either on the right intraparietal sulcus (IPS, a node of the dorsal attention network) or on the right temporo-parietal junction (TPJ, belonging to the ventral network). The spatially non-predictive peripheral cue was completely irrelevant for the task, and was presented at either 200 or 800 ms before target onset (stimulus onset asynchronies, SOAs). In these conditions, cues should induce facilitation at the short SOA and IOR at the long SOA (Chica et al., 2013). We observed that TMS interference on either the right IPS or the right TPJ prevented the expected occurrence of contralateral IOR at the long SOA, suggesting a causal implication of the caudal nodes of both attentional networks in exogenous attentional orienting. This result clearly indicates that parietal regions within both the dorsal and ventral networks in the right hemisphere play a pivotal role during exogenous orienting. TMS-mediated interference over these regions altered the time course of exogenous attention, even when cues were neither task-relevant nor set-relevant.

We followed up these results by using a target-target paradigm and an offline interference TMS design. TMS was used to lastingly interfere with either the right IPS or TPJ (Bourgeois et al., 2013a), or with their homologs in the left hemisphere (Bourgeois et al., 2013b). When participants maintained fixation at the center of the display (so called covert orienting, used in all the studies discussed above), interference on the right TPJ abolished IOR for right-sided, ipsilateral targets (mimicking results observed after right parietal damage or right fronto-parietal disconnection; Bourgeois et al., 2012; see also Bartolomeo et al., 1999), while interference on the right IPS abolished IOR bilaterally. Comparable stimulation of the left IPS or TPJ did not produce any significant modulation of the exogenous attentional effect (Bourgeois et al., 2013b), suggesting that hemispheric asymmetries in the control of visual attention are not confined to the ventral network (Nobre et al., 1997). Even though in a target-target design all stimuli are task-relevant and require a manual response, the feature that makes the trial valid or invalid is the target spatial location, which is task-irrelevant, because participants respond with the same keypress anytime a peripheral target is presented.

In conclusion, the above-mentioned TMS studies demonstrate that the right TPJ, a caudal node of the ventral attention network, plays a causal role in some aspects of exogenous orienting of spatial attention, even when the stimuli or their spatial features are not task relevant. This is at odds with the conclusion that mere bottom-up stimulus onset is insufficient to activate the ventral network, while the specific relationship between the characteristics of the external signal and the internal goals/expectations plays a pivotal role for the activation of this system (Macaluso and Doricchi, 2013). As it is evident from the scholarly review by Macaluso and Doricchi, research on attention has enormously benefitted from fMRI-based models, which have set up a comprehensive framework for theoretical discussion and inspired many new experiments. However, in the case of exogenous orienting and task-irrelevant information, activation of the ventral network might be so transient (with a time course of ~100–300 ms; Müller and Rabbitt, 1989) that fMRI designs might be insensitive to its effect. Making the stimuli task-relevant could enhance their brain-related activations and prolong their temporal course, which might make fMRI studies more sensitive to their related effects. We therefore suggest that complementary evidence from other techniques should be taken into account, especially when exploring the fast and short-lasting mechanisms of exogenous attentional orienting.

## Conflict of interest statement

The authors declare that the research was conducted in the absence of any commercial or financial relationships that could be construed as a potential conflict of interest.
